# Program evaluation of postgraduate obstetrics and gynecology training in Lao people’s democratic republic - using the CIPP model

**DOI:** 10.1186/s12909-023-04942-6

**Published:** 2024-01-09

**Authors:** Panima Chanthalangsy, Byung-Il Yeh, Seong Jin Choi, Yon Chul Park

**Affiliations:** 1 Department of Medical education, Yonsei University Wonju College of Medicine, Wonju, Gangwon-do Republic of Korea; 2 Department of OB-GYN, Yonsei University Wonju College of Medicine, Wonju, Gangwon-do Republic of Korea

**Keywords:** CIPP evaluation model, Curriculum evaluation, OB-GYN residency program evaluation, Program evaluation

## Abstract

**Background:**

The obstetrics and gynaecology (OB-GYN) residency training program in Lao People’s Democratic Republic (PDR) began in 2003 based on the Millennium Development Goals (MDGs) and ‘Reproductive, maternal, newborn, and child health interventions (RMNCH) strategies and action plan’. However, the training program had not been properly evaluated previously. The purpose of this study is to evaluate the current postgraduate OB-GYN residency training program in Lao PDR by using CIPP model to identify the current problems (the strengths and weaknesses) and suggest a future plan to promote continuous improvement.

**Method:**

The context, input, process, and product classification (CIPP) model was used to develop criteria and indicators. A mixed-methods approach was used for this study. To capture instructional material for quantitative analysis, a Google survey with 38 items and a t-test were used to determine a significant difference in responses between residents and lecturers (N = 120). Based on qualitative analysis, an in-depth interview was done (four questions based on study outcomes, including satisfaction, strengths and weaknesses, and future opportunities), and six interviews provided different viewpoints on the course. The SPSS software program was used to measure validity, with *p*-values = 0.05.

**Results:**

The overall average response rate was 97.5%. Two significant differences in program perspectives were revealed between lecturers and residents, difficulties in maintaining the course (professors 3.66 ± 1.03 and residents 3.27 ± 0.98, p = 0.04) and learning outcomes achieved (professors 3.57 ± 0.85 and residents 3.14 ± 0.95, p = 0.01 The overall average for the context part of the questionnaire was under 3.00, with the lowest scores for overlapped learning outcomes and difficulties in maintaining the course. The input part, lack of the classroom, skills lab and staff; the process part, lecturer to collect student opinions and the product part on learning outcomes.

**Conclusion:**

Curriculum improvement based on the program evaluation results, including regular evaluation and feedback, will advance the residency training program based on the RMNCH strategy and contribute to the promotion of maternal health in the Lao PDR.

**Supplementary Information:**

The online version contains supplementary material available at 10.1186/s12909-023-04942-6.

## Background

Maternal mortality has been identified as an important issue and the Lao People’s Democratic Republic (PDR) government attempts to address this in order to meet the Millennium Development Goals (MDGs) by 2015 [1]. The obstetrics and gynaecology (OB-GYN) program is designed to train medical professionals to provide services for the benefit of the community [2]. The Lao PDR government has embarked on a goal of achieving the MDGs by 2015, with a special focus on improving the maternal health service quality thus reducing maternal mortality. Obstetricians and gynaecologists serve the society through the provision of medical services [3]. At end of 2015, the new strategy replaced the UN Secretary-General’s 2011 Global Strategy for Women’s and Children’s Health. Problems of recruitment, allocation and supervision of skilled health workers; increased development of essential reproductive maternal new born and child health (RMNCH) including service coverage; preparation of environment facilities required to implement free maternal and child health policy to address inequality and inequity; empowerment of women and families in the community using supplied maternal and new born health services and adolescent pregnancy reduction will be critical [1].

The University of Health Sciences (UHS) is the only medical school and residency training facility in the Lao People’s Democratic Republic (Lao PDR) [4]. UHS adopted a competency-based training program in 2003, based on the Millennium Development Goals (MDGs) and Reproductive, Maternal, Newborn, and Child Health (RMNCH) strategies [1]. The program has eight components: medical knowledge, patient care and procedural skills, medical ethics and professionalism, interpersonal and communication skills, practice-based learning and improvement, systems-based practice, research skills and English skills. However, despite the program’s nearly two decades of operation, no evaluation has been conducted [5].

Context, input, process and product (CIPP) is one of the most widely used evaluation model. The CIPP model examines program conception and planning, including the achievement of learning objectives and goals. It also determines the length of a program, suitable for both learners and the specific purpose. CIPP is widely used to evaluate the initiatives and interactions and to identify its strengths and weaknesses in medical education research including graduate medical education [1, 6–8]. Moreover, this method is widely used not only evaluating programs in graduate medical education [9], but also in continuous professional development [10].

## Methods/Design

### Aims

The primary aim of this study is to evaluate the obstetrics and gynecology (OB-GYN) postgraduate training residency program at the University of Health Sciences (UHS) in Lao PDR using the CIPP model, including problem identification and recommendations for future plans.

### Study design

A mixed-methods approach was used in this research to evaluate the postgraduate training program for the OB-GYN residency course at the University of Health Sciences (UHS) in Lao PDR. A cross-sectional survey was conducted using the CIPP model, followed by in-depth qualitative interviews.

### Participants

**Fig. 1:**
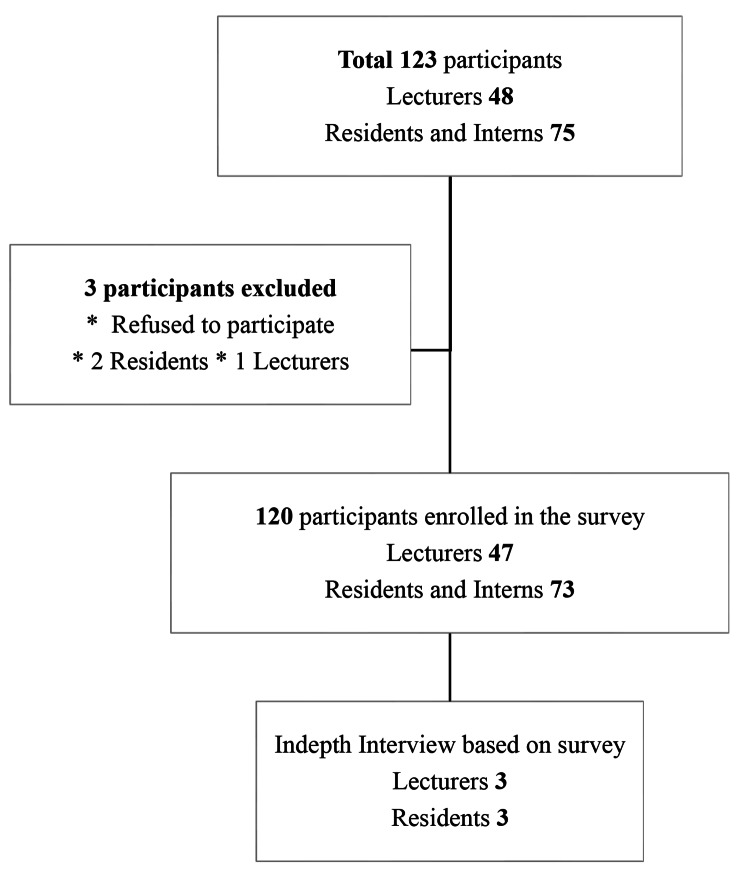
Study population

The UHS OB-GYN department consists of 75 residents and 48 faculty. A full investigation was conducted for the survey and interview, and representatives of each group were selected to participate. For the survey part, first-year to third-year residents, interns, OB-GYN level 1 specialists, and lecturers were surveyed in the research. A total of 123 participants (n = 123) were included in the study, as detailed in Fig. 1. All lecturers, residents, and interns who were enrolled in the class of 2022 participated in the study, and only individuals who agreed to participate in the survey were included.

The main course coordinator, lecturer, vice dean, and three OB-GYN residents (including two chief residents) participated in a 1- to 2-hour face-to-face interview via Zoom on a scheduled date. Audio transcripts of the interview were analyzed. Participants consented to the interview and signed permission forms in advance.

### Ethic approval

The study was approved by the Yonsei University Wonju Severance Christian Hospital. The study protocol conformed to the ethical guidelines of the 1975 Declaration of Helsinki [11], as reflected in prior approval by the Institutional Review Board of the Yonsei University Wonju College of Medicine (IRB No. 2022-0149-002). Informed consent was obtained from all research participants.

### Methodological approach

#### Questionnaire

The CIPP questions were organized through diverse article reviews in program evaluations, especially on the CIPP model and other countries’ program evaluations. The research team and a medical expert in Laos discussed and modified the survey questionnaire repeatedly. Content validity index testing was also performed, and three medical education experts reviewed the questionnaire in four key areas of the CIPP: context (program goal and barriers), input (inquiry on facilities and resources), process (teaching and learning), and product (learning goal achievement). A five-point Likert scale was used for all categories (5, strongly agree; 1, strongly disagree). Scores lower than 3.0 were considered insignificant. Additionally, one item was removed due to insignificance, and 38 survey items were finally interpreted using the online CIPP survey.

### Interview questions

The two-part interview questions consisted of 11 supplementary questions. The first part was general questions about OB-GYN course satisfaction, strengths and weaknesses, and future development. The second part consisted of specific questions based on the CIPP results. We asked 7 questions that scored an overall average below 3.00 on the questionnaire or showed different views between lecturers and residents. The context part questions included overlapped learning outcomes and the difficulties in maintaining the course. The input part asked questions about the classroom, skills lab, and staff. The process part asked lecturers to collect student opinions, and the product part clarified learning outcomes.

### Data collection

The study is conducted from 9 to 15 May. The study population was comprised of all lecturers, residents and interns who had enrolled in the OB-GYN residency course in Laos during the class of 2022, and a total of 120 participants are in this study.

The survey part is divided into two parts. Firstly, the characteristics identified seven parts as general factors including sex, age, current position, working experience and teaching experience and the OB-GYN subspecialists had been requested to answer the questionnaires. Secondly, in the CIPP part, 38 questionnaires were collected using Google forms. ZOOM was used to conduct the six recorded face-to-face interviews and the content analysis was performed online by asking the opinion of each category.

### Data analysis

The SPSS software program (version 25.0, IBM Corp., Armonk, NY, USA) was used to measure validity, and *p*-values ≤ 0.05 were considered statistically significant. Moreover, an independent samples t-test was completed to identify the difference between the two groups. Cronbach’s alpha was used to calculate the survey results and determine the reliability of the responses.

The content analysis approach was used to conduct the qualitative data analysis. The audio recordings of the interviews were converted into a written format, and each individual transcript was coded. Two researchers with experience in qualitative research read the transcribed data repeatedly to understand the data’s overall meaning, extracted words and sentences representing key thoughts or concepts related to the context, input, process, and product from the data’s content, and grouped sentences with similar meanings and topics together. Next, we grouped the topics into more abstract and meaningful topics based on their connections. During the analysis process, we attempted to increase the reliability and validity of the results by adjusting any differences in categories or concepts through continuous discussion and consultation between the two researchers.

## Results of the research

### General characteristics of the survey

The overall average response rate for the OB-GYN course for the 2022 class was 97.5% (123 respondents) of including 47 of all lecturers. In detail, 73 residents and interns responded. Two residents and interns and one lecturer declined to respond to the survey. Cronbach’s alpha was 0.979 for the total survey item and the data were are shown in Table 1.

**Table 1:** General Characteristics of the Survey Results

Variable	ALL		Lecturer		Resident and Intern	
	n = 120	%	N = 47	%	N = 73	%
**Sex**						
Male	41	34.17	16	13.33	25	20.83
Female	79	65.83	31	25.83	48	40.00
**Age**	34.08		39.09		30.85	
**Position**						
Lecturer	22	18.33	1	0.83	-	-
Clinical preceptor	21	17.50	21	17.50	-	-
Resident	60	50.00	-	-	56	46.67
Intern	17	14.17	-	-	16	13.33
**Working experience years**	-	-	10.0	-	-	-
**Main sub-specialty**						
General obstetrics and gynaecology	-	-	36	30.00	-	-
Oncologic gynaecology	-	-	5	4.17	-	-
Urogynaecology	-	-	0	0.00	-	-
Minimally invasive gynaecology Surgery	-	-	-	-	-	-
Maternal foetal medicine	-	-	4	3.33	-	-
Reproductive medicine	-	-	1	0.83	-	-
Administration and research	-	-	1	0.83	-	-
No sub-specialty	-	-	0	0.00	-	-
**Teaching experiences**	-	-	5.68	-	-	-

*Data are presented as mean ± SD

### Program evaluation using the CIPP model

The survey results showed that the product part had the greatest number of satisfactory remarks in the evaluation (3.46 ± 0.83). Lecturers (3.56 ± 0.73) and residents (3.40 ± 0.89) were satisfied with the current OB-GYN program outcome and set outcome, which is also in line with the RMNCH strategy development plan in 2016–2025 (*p*-value = 0.29). All data are shown in Table 2, and the results of the four sections of the CIPP model are shown in Table 3.

**Table 2:** Survey Results of the Program Evaluation Using the CIPP Model

	All	Lecturer	Resident & Intern	*p*-value
Context	3.39 ± 0.79	3.47 ± 0.87	3.34 ± 0.79	0.37
Input	3.22 ± 0.78	3.22 ± 0.76	3.22 ± 0.80	0.98
Process	3.32 ± 0.75	3.42 ± 0.62	3.25 ± 0.82	0.22
Product	3.46 ± 0.83	3.56 ± 0.73	3.40 ± 0.89	0.29

*T-test was used

**Table 3:** CIPP Answers Comparing Lecturers and Residents

Questions	All (N = 120)	Lecturer (N = 47)	Resident and Intern (N = 73)	*p*-value
**1. Context evaluation**				
Training goals and outcomes are clearly defined	3.48 ± 1.02	3.64 ± 0.97	3.37 ± 1.05	0.16
Trainees understand the training goals	3.39 ± 0.93	3.57 ± 0.93	3.27 ± 0.92	0.08
The learning goals are set for each year of residency	3.59 ± 0.91	3.66 ± 0.87	3.55 ± 0.94	0.51
Course orientation was conducted before starting	3.86 ± 1.09	3.79 ± 1.14	3.90 ± 1.06	0.56
Pre-survey was done before starting	3.19 ± 1.01	3.23 ± 0.96	3.16 ± 1.04	0.71
Surveyed students’ opinions	3.22 ± 0.96	3.15 ± 1.04	3.26 ± 0.91	0.53
Difficulty in maintaining the course	3.43 ± 1.01	3.66 ± 1.03	3.27 ± 0.98	0.04*
Content was appropriate	3.37 ± 0.96	3.53 ± 1.00	3.26 ± 0.93	0.13
Lecturers and residents are aware of the program’s goals	3.43 ± 0.98	3.53 ± 0.98	3.37 ± 0.98	0.37
** The learning outcomes of the subjects did not overlap**	**2.99 ± 0.95**	**2.98 ± 0.99**	**3.00 ± 0.93**	0.90
**2. Input evaluation**				
** Appropriate and enough classrooms**	**2.99 ± 1.06**	**2.98 ± 1.07**	**3.05 ± 1.05**	0.41
Appropriate and enough study materials	3.13 ± 1.09	3.04 ± 1.04	3.19 ± 1.13	0.46
** Appropriate and enough skills lab**	**2.88 ± 1.09**	**2.87 ± 1.04**	**2.89 ± 1.14**	0.93
The lecturer using a variety of teaching methods	3.28 ± 1.00	3.30 ± 1.00	3.27 ± 1.00	0.89
The syllabus (training plan) was given on time	3.26 ± 0.95	3.34 ± 0.98	3.21 ± 0.93	0.44
The school provides faculty development program	3.47 ± 0.95	3.53 ± 0.95	3.42 ± 0.96	0.54
Lecturers participate in the faculty development program	3.44 ± 0.96	3.55 ± 0.95	3.37 ± 0.97	0.30
** Enough staff or assistants**	3.04 ± 0.95	**2.96 ± 0.91**	3.10 ± 0.97	0.43
The teaching method was run conforming with the syllabus	3.47 ± 0.95	3.49 ± 0.95	3.45 ± 0.96	0.83
**3. Process evaluation**				
Teaching methods suited with the learning outcomes	3.36 ± 0.90	3.40 ± 0.85	3.33 ± 0.93	0.65
Students can easily communicate or see the faculty	3.38 ± 0.99	3.45 ± 0.97	3.34 ± 1.00	0.57
The lecturers prepare the lessons in advance	3.39 ± 0.97	3.51 ± 0.91	3.32 ± 1.01	0.28
Time schedule changes a lot	3.23 ± 0.88	3.21 ± 0.86	3.25 ± 0.89	0.83
Lecturers are using content that engages students	3.31 ± 0.92	3.38 ± 0.77	3.26 ± 1.00	0.47
Lecturers provide comments or suggestions	3.37 ± 1.00	3.57 ± 0.83	3.23 ± 1.09	0.05
** Lecturers collect student opinions**	3.03 ± 0.99	3.15 ± 0.83	**2.95 ± 1.08**	0.27
Lecturers always attend the resident presentation	3.14 ± 0.94	3.30 ± 0.83	3.04 ± 0.99	0.14
Lecturers conduct individual coaching regarding procedure or surgery	3.52 ± 0.94	3.68 ± 0.86	3.41 ± 0.98	0.12
Lecturers supervise the clinical research activities	3.40 ± 0.87	3.47 ± 0.78	3.36 ± 0.93	0.49
Lecturers conduct clinical teaching	3.34 ± 0.92	3.49 ± 0.75	3.25 ± 1.01	0.13
**4. Product evaluation**				
** The learning outcomes are achieved**	3.31 ± 0.93	3.57 ± 0.85	3.14 ± 0.95	0.01*
The learning outcomes help improve the RMNCH strategy	3.62 ± 0.98	3.70 ± 0.88	3.56 ± 1.04	0.44
Evaluation survey was collected after the course	3.08 ± 0.99	3.04 ± 0.83	3.11 ± 1.09	0.70
This course was an overall success	3.34 ± 0.90	3.49 ± 0.83	3.25 ± 0.94	0.15
Will this course substances in the next year	3.47 ± 0.90	3.62 ± 0.80	3.37 ± 0.95	0.14
Satisfied regarding the OB-GYN program	3.45 ± 0.98	3.62 ± 0.80	3.34 ± 1.07	0.11
Unexpected positive outcomes	3.51 ± 1.04	3.62 ± 0.95	3.44 ± 1.09	0.35
The course is beneficial in growing your career in the future	3.94 ± 1.11	3.85 ± 0.96	4.00 ± 1.20	0.47

* *p*-values < 0.05

**In the content evaluation.** Residents and lecturers provided a negative review on the question “The learning outcomes of the subjects did not overlap.” On the contrary, both residents and lecturers provided a strongly positive review on the statement “An orientation was conducted before beginning the OB-GYN program,” which is a helpful first step for residents to gain a clear awareness of the study preparations. Moreover, the feedback and faculty training programs are currently deemed to be the best methods to enhance faculty knowledge and skills.

**In the input evaluation**. Most lecturers and residents thought that there was “difficulty maintaining the course.” They also had a negative view of the “insufficient classroom and no skills lab.” However, only lecturers thought that there were not “enough staff or assistants,” but this was statistically insignificant.

**In the process evaluation.** Both groups had a “very positive impression” of the close relationships between residents and lecturers, which are “very beneficial” for direct feedback and exchange of ideas face-to-face. However, students gave negative feedback on the statement “Lecturers collect student opinions,” but this was also statistically insignificant.

**In the product evaluation.** By implementing the new RMNCH strategy, both groups were satisfied with all aspects of the OB-GYN courses, which helped them expand their careers in the near future. Most of them mentioned that education will enable residents to enhance patient lives as skilled physicians. In conclusion, they expressed the opinion that the current OB-GYN program meets RMNCH criteria and suits their needs.

There were only two significantly different opinions on the CIPP results. First, the lecturers had difficulty maintaining the course (P = 0.04); moreover, the residents and lecturers should consider more on the achievement of the learning outcomes (P = 0.01). The degree of satisfaction was higher in the lecturers than in the residents.

### Qualitative results

#### Depth interview part

Both lecturers and residents were generally satisfied with the current course, as detailed in Table 4. According to Lecturer (A), “The practical hours were up to 70% and the theory was 30% compared with the previous program, where theory was 60% and practice was 40%.” Most of the residents also expressed their satisfaction with the curriculum: “We are satisfied with the program, particularly the practice section since we can practice with a real patient nearly 100% of the time under supervision.” All participants highlighted the strengths of the course: “We practice and deal with an actual patient approximately 100% of the time.” Most of the residents said, “The learning activities could help us learn a lot” and “The lessons are up-to-date and many scientific papers are on our website which help us learn and shows us how to diagnose a patient.”

**Table 4:** Demographics of the Interview Participants

Participants	Age	Sex	Position	Sub-specialty
A	56	Male	Vice dean of the faculty of medicine	Maternal foetal medicine
B	41	Male	Main course Coordinator	Level I specialist for OB-GYN
C	44	Male	Lecturer	Oncologic gynaecology
D	29	Female	2nd year resident	N/A
E	30	Female	1st year resident	N/A
F	29	Female	2nd year resident	N/A

Regarding weaknesses in the OB-GYN course, both groups agreed that “The resident’s acknowledgement and English skills are totally different” due to their place of residence before continuing the course. Most of them need to improve their reading focus and their English skills. Moreover, “The residents need to have more lecture time” which was opposite for the lecturers who said “they need to reduce lectures.” Therefore, “the budget” as well as “the insufficient classroom and Internet” are limitations.

## Context interview

There was some difference of opinion between the lecturers and the residents regarding the overlap in learning outcomes. The lecturer said that the content is continuous from basic to practice, and that residents may learn technical differences in the four main hospitals. A resident said that they have been learning the content for a time, but that they have to learn it twice and that the class needs to be separated between residents and interns because residents should focus more deeply than interns. Faculty complained of lack of time and excessive work in maintaining the course. Lecturer (A) said that “Time consuming is difficult because the lecture has work overload in hand.” And Lecturer (C) complained that “We have discussion in a monthly faculty meeting, preceptor meeting, resident meeting every 3 months.” The residents also participated in those meetings.

**Input Interview** Both lecturers and residents complained about the resources, including classrooms, skill labs, and equipment. Lecturer (C) said, “We have a skill lab, but the equipment is not sufficient and the room is now under construction, so residents can’t use it.” Resident (C) said, “We never practice in the skill lab; we only practice with patients in the hospital.” Regarding the number of staff, both lecturers and residents agreed that more are needed, but they also said that the current staff and assistants are doing their best. Lecturer (B) said, “The number of residents is greater than the number of staff, so there is a shortage of staff.” Resident (B) said, “I think there is a shortage of staff, and the coordinator is also not enough. But they are really doing a good job.”

**Process Interview** For collecting the opinions, both groups thought they were doing well, but they were not collecting the residents’ opinions well. Lecturer (B) said, “We use the feedback from residents every three months.” And Resident (B) said, “I’m not sure about faculty meetings, but we only have feedback practice in each hospital after our rotation by using a Google form.” For achieving the learning outcomes, both groups were satisfied, but contrary to the questionnaire results, we could not find any differences. Lecturer (A) said, “We can see that we succeeded in reducing maternal mortality since we started the OB-GYN course in 2003.” And Resident (C) said, “The course really benefits my needs. I can see, give treatment to, and take care of patients in the real workplace.”

**Product Interview** For achieving the learning outcomes, both groups were satisfied, but contrary to the questionnaire results, we could not find any differences. Lecturer (A) said, “We can see that we succeeded in reducing maternal mortality since we started the OB-GYN course in 2003.” Resident (C) also said, “The course really benefits my needs. I can see, give treatment to, and take care of patients in the real workplace.

## Discussion of the research

This is the first nationwide evaluation of Lao PDR’s OB-GYN residency program. Although there are still certain problems to overcome, the findings show that the overall aims have been met to a considerable degree. The lecturers and residents all agree that this training program’s major difficulty is meeting the standards.

**In the content evaluation.** Residents may recognize the significance of their training, highlighting the need for identifying learners’ needs and creating program objectives and assessments early in the starting period. Similar to previous research [12], the educational purpose provided clearly in the syllabus will help represent the students’ needs and level with the class, helping them reach curricular objectives. According to [13], teachers’ excitement for education should be fostered in all courses, as indicated by students’ learning successes. Lecturers should not overreach educational content. In fact, the faculty training program is the greatest method to develop faculty knowledge and abilities.

**In the input evaluation**, we found that it was difficult to borrow or reserve a room due to space shortage. If there is none available, the class will have to be extended or switched to an online teaching platform such as Google Classroom or Zoom meeting. When the coronavirus disease 2019 (COVID-19) broke out, online classes increased [14], which showed that students tend to express their thoughts during online class sessions and are more successful when the lecturer acts as a guide rather than a leader. Presently, educators might utilize Zoom instead of a classroom; however, they could not practice essential skills. Furthermore, the college of medicine in Laos plans to create a new simulation center and objective structured clinical examination (OSCE) room for licensing exams in the next 3 years. We agreed that this proposal is in keeping with residents’ needs and will benefit all residency programs in the near future.

**In the process evaluation**, both residents and lecturers agree that hands-on teaching is the most helpful way for learning and getting real feedback. Exploring with real patients and providing individual coaching are well-known and successful methods adopted in the Lao OB-GYN residency program. A research study on pedagogy teaching [15], elucidated that one of the most significant components of teaching is open communication, which begins with a full description of the lesson plan, educational objectives, procedures, assessment, criteria, and attitudinal guidelines. Pre-procedure talks before beginning work enable lecturers to check students’ degree of classifier knowledge and modify learning to their needs [16]. Recognizing the roles that lecturers need to pay more attention to students, we believe that open communication can help in gaining real feedback from lecturers and students. Additionally, students need to prepare to enhance their professional skills and understand procedures as well as technical surgeries. Thus, feedback is an important part of informing resident progress, seeing their improvement, and engaging in appropriate learning activities [17].

**In the product evaluation**, the new RMNCH strategy generally placed the OB-GYN program outcomes-enhancing priorities on the maternal death monitoring program by offering treatment guidelines, strategies, recommendations, and training to prevent maternal and fetal deaths. The lecturer believes that they can help in enhancing future specialties. Hands-on practice and resident follow-up allow residents to implement what they have learned throughout the training, thereby enhancing the RMNCH strategy [1, 18].

According to “Lack of access to learning resources,” the standard handbook did not contain Laos’ language. The course uses a Thai-English textbook. Moreover, e-learning was advised to be in line with this rapidly changing environment [19]. This will help address the lack of knowledge, study tools, technology, and teaching personnel. In a fast-paced world, e-learning can be advantageous if designed and incorporated into a student’s schedule efficiently [20]. Since students can acquire online resources and e-learning via the internet, these may help residents transition to the new method of self-learning. However, free internet access needs to be considered in the workplace area.

Most of the residents and lecturers mentioned “The class did not conduct surveys to gather student opinions,” and over half of the lecturers had never gathered resident feedback before. Residents’ opinions are essential for improving the quality of teaching. A study from Lewisson said that student ratings were one technique for faculty growth in teaching and learning after each semester [21]. This includes formally classifying rotations. According to a recent result, lecturers should begin gathering feedback from residents to assess their own education and develop methods for continuous quality improvement in the next few years. The OB-GYN program includes information on how to prevent these issues.

“Lack of staff” was one problem in the program. Several articles showed how to determine the optimal number of faculty needed to compare with resident number: less than 25 trainees in a program need one program staff, or more than 50 trainees should have three staff attending to them [22]. Depending on the current situation, the director of the program assigned a “clinical preceptor” to assist in following and teaching the residents during their rotation. This puts a clinical preceptor as part of the educational system instead of increasing the number of staff, which is beneficial for the curriculum.

Moreover, “The residents wanted more lecture sessions” without a clear explanation or approbation from the lecturers. In general, lecturers may offer a quick explanation or set them up to study in small peer-to-peer groups to improve learning and peer interaction. More weekly class time on average tends to enhance academic success [23]. Flipped or small-group teachings may benefit from learner-specific themes. Giles and colleagues found that medical students remembered best between 15 and 30 min, whereas the first 15 min had the worst retention. A quick overview or a 15- to 30-minute lecture would boost residents’ knowledge and joy of learning [24]. Also, our findings concur with Giles and colleagues’ recommendation that most OB-GYN residencies need more short summaries or lectures to enhance understanding.

In conjunction with “the course’s outcome”, according to a resident, several of the lecture outcomes overlapped, but the lecturers had a distinct perspective. The lecturers mentioned that the topics were planned out with continuous theory lectures from the beginning of the class until the rotations. Some may appear to “overlap” since each educator from four separate hospitals uses different guidelines; thus, pupils may receive diverse approaches and skills. This should be a question at faculty meetings and residency talks to emphasize the importance of their training. Furthermore, The Accreditation Council for Graduate Medical Education (ACGME) standard-setting guidelines may help resolve the overlap problem and advise residents to improve their performance and learning [2]. One respondent advised separating each lecturer’s delivery of standard material to eliminate topic overlap.

While conducting the in-depth interview, replies with higher and lower CIPP results in each section of the question were included. Thus, to determine what is lacking in the curricula, we compared both lecturers’ and residents’ viewpoints in the survey. The short summary lists the strengths and weaknesses of the problem and suggestions regarding the current OB-GYN program, including the reasons. The suggestions are shown in Table 5.

**Table 5:** A Short Summary List of Problems and Suggestions Regarding the Current OB-GYN Program, Including Reasons

Problem	Suggestion	What to do
• The outcome of the class had overlapped	• Clearly separate delivery outcomes for each workplace	• Pre-meeting with rotation clerkship manager
• Lack of staff and lecturers	• Providing clinical preceptors to guide residents in the rotation	• Setting standard roles for clinical preceptors
• Insufficient classroom and skills lab	• Online learning	• Faculty development about online learning and plan for a future classroom and skills lab
• Lack of collecting resident’s opinion	• Provide information regarding the benefits of student feedback	• Regular survey for quality teaching improvement
• Resident’s English skills are insufficient	• Increase English class	• Make quarter for English class
• Lack of funding or budget	• Contact NGO or government support	• Making future plans and meetings with stakeholders

This study has a few limitations. First, only content validation was done, and no face-to-face interviews were conducted due to the COVID-19 pandemic. Moreover, the number of interview groups was small (6 people), and we could not get the opinion of all stakeholders, including all staff and coworkers who participated in the educational system. Despite these limitations, this was the first evaluation of the Lao PDR’s OB-GYN program at the UHS, and the response rate of the participants in the survey was 97.5% (n = 120). Also, the interviewee had a comparative perspective between the two groups: lecturers and residents, which made our findings valuable. Even if there are still certain challenges to be overcome, the data suggest that the program’s main goal has been achieved to a significant degree.

## Conclusion

The curriculum needs to be evaluated regularly to improve the OB-GYN residency program. The course director needs to modify and ensure time management to enhance the activities of learning, such as small lectures to help students learn deeply. Although students should study on their own, lectures require additional learning modules. With the participants’ responses in our study, we can conclude that our lecturers and residents are positively satisfied with the current OB-GYN residency program. Therefore, the most important task is to raise lecturers’ and residents’ awareness of the program’s outcome. The program can successfully achieve most of the training objectives that suit the RMNCH strategy.

### Electronic supplementary material

Below is the link to the electronic supplementary material.

Supplementary Material 1

## Data Availability

All data and materials can be made available to the journal on request. Request for data can be submitted to the corresponding author by email provided below. iamyonchul@yonsei.ac.kr.
