# Supplementary material for: Program evaluation of postgraduate obstetrics and gynecology training in Lao people’s democratic republic - using the CIPP model
Source: BMC Med Educ. 2024 Jan 9;24:44. doi: 10.1186/s12909-023-04942-6 (PMC10775444; doi:10.1186/s12909-023-04942-6)
Supplement: Supplementary file 1 — Supplementary Material 1 [file 12909_2023_4942_MOESM1_ESM.docx]

**Supplementary Information**

Survey for Program Evaluation of postgraduate training program OB-GYN in University of Health Sciences Lao PDR

**I. Socio-demographic data**

**Instruction:** please tick in the box and fill in the blank where appropriate.

1. Gender:

☐ Male

☐ Female

1. Age: ________ years 󠄀
2. What is your work or education positions?

☐ Interns

☐ Residents

☐ Lecturers

☐ Clinical Preceptor

1. What is your current status?

☐ Interns

☐ Residents

☐ Lecturers

1. How long have you been working in OB-GYN fields? (For lecturers only)

_________ years

1. What is your main major or primary subspecialty in OB-GYN fields?

☐ General Obstetrics and Gynecology

☐ Oncologic Gynecology

☐ Urogynecology

☐ Minimally Invasive Gynecology Surgery

☐ Maternal Fetal Medicine

☐ Reproductive Medicine

☐ Administration and Research

☐ No subspecialty

1. How long have you been teaching for OB-GYN course? (For lecturers only)

_________ years

**II. Question-based on the CIPP model**

- The questionnaires using a five-point rating scale follows: 1 (strongly disagree) to 5 (strongly agree) following with CIPP model.

1. Strongly disagree.
2. Disagree.
3. Neither agree nor disagree.
4. Agree.
5. Strongly agree.

| **Items** | **Questions** | | Strongly disagree | Disagree | Neither agree nor disagree | Agree | Strongly agree |
| --- | --- | --- | --- | --- | --- | --- | --- |
| **Context** | **Are the program being meet the goal?** | 1. Do you think the training goals and outcomes of this program are clearly define? |  |  |  |  |  |
|  |  | 1. Do you think the trainees understanding the training goals and expected outcomes? |  |  |  |  |  |
|  |  | 1. Are The learning goals set for each year of residency? (Year 1 , 2, 3) |  |  |  |  |  |
|  |  | 1. Does the course orientation before starting ? |  |  |  |  |  |
|  |  | 1. Is there pre-survey before begin the class? |  |  |  |  |  |
|  |  | 6. Were these curriculum used to survey students' opinions? |  |  |  |  |  |
|  |  | 7. Did the faculty discuss the difficulties of maintaining the course? |  |  |  |  |  |
|  | **What are some of the barriers to the program's successful implementation?** | 8. Are the learning outcomes and lecture structure and content appropriate? |  |  |  |  |  |
|  |  | 9. Are the lecturers and residents aware of the program's goals/objectives? |  |  |  |  |  |
|  |  | 1. Are the learning outcomes of the subject not overlapped? |  |  |  |  |  |
| **Input** | **Facilities (space for learning, classroom environment, equipment’s for teaching and learning...)** | 11. Is there appropriate and enough classrooms or meeting room in the department? |  |  |  |  |  |
|  |  | 12. Is there appropriate and enough study materials and supplies in the department? (Library, Notebook, LCD, Audiovisual equipments...) |  |  |  |  |  |
|  |  | 13. Is there appropriate and enough skill lab or practice place in the department? |  |  |  |  |  |
|  | **Resources (supply of money, Staff, coordinators or lecturers, Quality )** | 14. Are the lecturer using variety of teaching methods in class? (Small group teaching, Questions and answers part during class...) |  |  |  |  |  |
|  |  | 15. Were the syllabus (training plan) given on time? |  |  |  |  |  |
|  |  | 16. Does the school provide faculty development program? |  |  |  |  |  |
|  |  | 17. Does the lecturers participant in faculty development program? |  |  |  |  |  |
|  |  | 18. Is there enough staff or assistants to help? |  |  |  |  |  |
|  |  | 19. Was the teaching method (Ex: Case presentation, Journal club, Lecture...) run in order to conform with the syllabus (training plan)? |  |  |  |  |  |
| **Process** | **Teaching and learning** | 20. Was the teaching method (Ex: Case presentation, Journal club, Lecture...) suit with the learning outcomes? |  |  |  |  |  |
|  |  | 21. Can students easily communicate or see with faculty? |  |  |  |  |  |
|  |  | 1. Does the Lecturers prepare lessons (For Case presentation, Journal club, Lecture...) in advance? |  |  |  |  |  |
|  |  | 23. Does the time schedule had not been changed a lot? |  |  |  |  |  |
|  |  | 24. Are the lecturers using content that engages students to study? |  |  |  |  |  |
|  |  | 25. Does the lecturers provide the comment or suggestion on bedside teaching journal club or case presentation? |  |  |  |  |  |
|  |  | 26. Does the lecturer actually collect the opinions of the students after class? |  |  |  |  |  |
|  |  | 27. Does the Lecturers always attend the Resident presentation? |  |  |  |  |  |
|  |  | 1. Does the lecturers conduct individual coaching in procedure or surgery? |  |  |  |  |  |
|  |  | 1. Does the lecturers supervise the clinical research activities? |  |  |  |  |  |
|  |  | 1. Does the lecturers provide the clinical teaching (bedside teaching , case cased discussion)? |  |  |  |  |  |
| **Product** | **Have the set learning goals been achieved?** | 31. Has the set learning outcomes been achieved? |  |  |  |  |  |
|  |  | 32. Did the learning outcomes help to improve the reproductive health status and reduce maternal, neonatal, and child mortality and morbidity including malnutrition in Lao PDR?  **The goal of the RMNCH Strategy & action plan 2016-2025 expand below. |  |  |  |  |  |
|  |  | 33. Are there evaluations survey used to collect after the course? |  |  |  |  |  |
|  |  | 34. Do you think this course was a success in overall? |  |  |  |  |  |
|  |  | 35. Is this course will substances in the next year? |  |  |  |  |  |
|  |  | 36. Are you satisfy about the OBGYN program? |  |  |  |  |  |
|  |  | 37. Have there been any unexpected positive outcomes? |  |  |  |  |  |
|  |  | 38. Have there been any unexpected negative outcomes? |  |  |  |  |  |
|  |  | 39. Are the courses beneficial in growing your career in the future? |  |  |  |  |  |

**Questionnaires for Depth Interview part**

1. Are you satisfied with the OB-GYN course? Which part do you like most and which part do you dislike? Why?
2. Do you think the program has any strengths and weaknesses? Give me some examples, please.
3. These eight questions based on the questionnaire of survey results: Could you explain your opinion each one please.

| Content | 1. Do you think are the learning outcome not overlapped? For example, the content of some class is similar or overlapped, what do you think about the contents of teaching in each class? 2. Did the faculty discuss the difficulties in maintaining the course? And how? |
| --- | --- |
| Input | 1. Is there enough classroom or meeting room for running the course? If not, how could you fix this problem? 2. Is there appropriate and enough skill lap or practice place in the OB-GYN department? 3. Is there enough staff or assistance to help? For example: In maintaining the class or connect with lecturer and resident. |
| Process | 1. Does the lecturer collect the opinion of the student after finishing the class? |
| Product | 1. Do you think the learning outcome in OB-GYN course had been achieved? and how ? |

1. Future development of the OB-GYN course.

- Do you have any suggestion or opinion that help how to improve the OB-GYN course? What could we do to improve the OB-GYN residency program? Please feel free to describe.

**Calculating Content Validity Index**

| No | Expert. 1 | Expert. 2 | Expert. 3 | Standard Division | Average | CVI |
| --- | --- | --- | --- | --- | --- | --- |
| Item 1 | 5.00 | 5.00 | 5.00 | 0.00 | 5.00 | 1.00 |
| Item 2 | 4.00 | 5.00 | 5.00 | 0.47 | 4.66 | 0.93 |
| Item 3 | 5.00 | 3.00 | 5.00 | 0.94 | 4.33 | 0.86 |
| Item 4 | 5.00 | 3.00 | 5.00 | 0.94 | 4.33 | 0.86 |
| Item 5 | 5.00 | 3.00 | 4.00 | 0.81 | 4.00 | 0.8 |
| Item 6 | 4.00 | 2.00 | 4.00 | 0.94 | 3.33 | 0.66 |
| Item 7 | 3.00 | 4.00 | 3.00 | 0.47 | 3.33 | 0.66 |
| Item 8 | 5.00 | 3.00 | 4.00 | 0.82 | 4.00 | 0.80 |
| Item 9 | 4.00 | 4.00 | 5.00 | 0.47 | 4.33 | 0.86 |
| Item 10 | 5.00 | 5.00 | 5.00 | 0.00 | 5.00 | 1.00 |
| Item 11 | 5.00 | 5.00 | 5.00 | 0.00 | 5.00 | 1.00 |
| Item 12 | 5.00 | 5.00 | 4.00 | 0.47 | 4.66 | 0.93 |
| Item 13 | 5.00 | 5.00 | 5.00 | 0.00 | 5.00 | 1.00 |
| Item 14 | 5.00 | 5.00 | 5.00 | 0.00 | 5.00 | 1.00 |
| Item 15 | 5.00 | 5.00 | 5.00 | 0.00 | 5.00 | 1.00 |
| Item 16 | 5.00 | 5.00 | 5.00 | 0.00 | 5.00 | 1.00 |
| Item 17 | 5.00 | 5.00 | 5.00 | 0.00 | 5.00 | 1.00 |
| Item 18 | 5.00 | 3.00 | 5.00 | 0.94 | 4.33 | 0.86 |
| Item 19 | 4.00 | 3.00 | 5.00 | 0.81 | 4.00 | 0.8 |
| Item 20 | 5.00 | 5.00 | 5.00 | 0.00 | 5.00 | 1.00 |
| Item 21 | 5.00 | 4.00 | 5.00 | 0.47 | 4.66 | 0.93 |
| Item 22 | 5.00 | 5.00 | 5.00 | 0.00 | 5.00 | 1.00 |
| Item 23 | 5.00 | 5.00 | 5.00 | 0.00 | 5.00 | 1.00 |
| Item 24 | 5.00 | 5.00 | 5.00 | 0.00 | 5.00 | 1.00 |
| Item 25 | 5.00 | 3.00 | 4.00 | 0.81 | 4.00 | 0.8 |
| Item 26 | 5.00 | 5.00 | 5.00 | 0.00 | 5.00 | 1.00 |
| Item 27 | 5.00 | 5.00 | 5.00 | 0.00 | 5.00 | 1.00 |
| Item 28 | 5.00 | 5.00 | 5.00 | 0.00 | 5.00 | 1.00 |
| Item 29 | 4.00 | 5.00 | 5.00 | 0.47 | 4.66 | 0.93 |
| Item 30 | 5.00 | 5.00 | 5.00 | 0.00 | 5.00 | 1.00 |
| Item 31 | 5.00 | 5.00 | 5.00 | 0.00 | 5.00 | 1.00 |
| Item 32 | 5.00 | 5.00 | 5.00 | 0.00 | 5.00 | .001 |
| Item 33 | 5.00 | 5.00 | 5.00 | 0.00 | 5.00 | 1.00 |
| Item 34 | 5.00 | 4.00 | 3.00 | 1.00 | 4.00 | 0.80 |
| Item 35 | 5.00 | 3.00 | 5.00 | 0.94 | 4.33 | 0.86 |
| Item 36 | 5.00 | 4.00 | 5.00 | 0.47 | 4.66 | 0.93 |
| Item 37 | 5.00 | 4.00 | 5.00 | 0.47 | 4.66 | 0.93 |
| Item 38 | 4.00 | 4.00 | 5.00 | 0.47 | 4.33 | 0.86 |
| Item 39 | 5.00 | 3.00 | 3.00 | 0.94 | 3.66 | 0.73 |
| Item 40 | 5.00 | 3.00 | 5.00 | 0.94 | 4.33 | 0.86 |
| Item 41 | 5.00 | 3.00 | 4.00 | 0.81 | 4.00 | 0.80 |
| Item 42 | 5.00 | 5.00 | 5.00 | 0.00 | 5.00 | 1.00 |

**Analyzing Compare Between Lecturers’ and Residents’ Perspective in Depth Interview art**

| **Themes** | **Categories** | **Sub-Categories** | **Lecturers (N=3)** | **Residents (N=3)** |
| --- | --- | --- | --- | --- |
| **The evaluation of current OB-GYN residency training course** | **Satisfied with the OB-GYN course** | **Satisfied** | **Lecturer 1.** “We can improve a number of the lecturer and increased of the practical hours up to 70%, lecture 20% and final project 10% and we can control and teaching on High-risk pregnancy in 4 main hospitals.”  **Lecturer 2.** “We updated and followed the guideline by changed from teacher centered to student centered.”  **Lecturer 3.** “This course can use in real situation, resident who completed this course are able to do or prevent the emergency in OB-GYN in whole country, also reduce the dead of maternal and childbirth.” | **Resident 1.** “I’m satisfied about the program especially the practice part because we are able to practice with real patient almost 100% under supervisor. Because we can practice by our self we can remember and understand all the step we have done clearly.”  **Resident 2.** “The set-up schedule is good. In every practice we have doing under supervisor of teacher, they give us a right to do with real patient.”  **Resident 3.** “I’m satisfied about the program especially the practice part, I am able follow up the patient and report to my supervisor.” |
|  |  | **Unsatisfied** | **Lecturer 1.** “Classroom is not enough for conduct a small group of teaching, we don’t have high therapy technology. Second is no dormitory for resident to stay.”  **Lecturer 2.** “Resident’s knowledge level is not equal, second is we don’t have one place rotation for practice, we have to rotate in four main hospitals and each system are difference.”  **Lecturer 3.** “The implementation according to the curriculum can’t follow 100% For example we don’t have one place rotation or university hospital.” | **Resident 1.** “The lecture time or learning in lecture room is less than I expect. We need more lecture or a short lecture to make clearly understand of the lesson, because the real practice and theory is really different.”  **Resident 2.** “We want more lecture and more explanations from the teacher in the form of brief lectures. Second, about the feedback in hospital, we need in real-time feedback.”  **Resident 3.** “I think a lecture time is not enough based to residency knowledge, there is some lesson that we couldn't understand all. It would be better to have more lecture time.” |
|  | **Strengths and weaknesses** | **Strengths** | **Lecturer 1.** “We increase sending our lecture aboard for sub-specialty.”  Lecturer 2. “We have significant number of patients and resident can practice and working with real patient 100%.”  **Lecturer 3.** “Residents are able to get experience working and practice with real patients nearly 100% under the supervision, and each hospital has a significant number of patients.” | **Resident 1.** “We got an experience working in the hospital and happy that we are able to practice with real patients, the teacher gives a right to us that we can take care of the patient. Second, we have a lecture time every Wednesday in the hospital and journal club presentation every Friday, these can improve knowledge a lot for us, we also have update lesson a lot in our website.”  **Resident 2.** “We have a chief resident good contact with coordinator. Second, we can practice with real patient almost 100% under supervisor, we can gain more than knowledge.”  Resident 3. “We can practice under supervisor in rotation and the main coordinator was good because we can communicate what we want and difficulty to him.” |
|  |  | **Weakness** | **Lecturer 1.** “The budget is limited.’  **Lecturer 2.** “Resident knowledge is not equal, we don’t have university hospital, staff is not enough comparing with student number.”  **Lecturer 3.** “Time management in curriculum is short compare with another country, second lecturer are work overload, they can’t follow all resident and we can miss some of weakness. Third, we don’t have our own study resource such as the website or textbook”. | **Resident 1.** “Each resident knowledge is not equal; we have difficulty to working in each hospital because each place using a difference guideline.’ Second, even we have lecture two times a week but it’s not sufficiently for us. Third, we only reading a journal in English also presentation slide. some of us has limitation in English skill.”  **Resident 2.** “First is about the lecture time is not enough. Second, we don’t have a fix classroom in the department. Third, we don’t have internet for using in school and workplace area, we have to use our own data.”  **Resident 3.** “My English is not good enough to reading textbook in English, and I need more lecture by lecturers.” |
|  | **How to improve the OB-GYN course** | **Suggestion or opinion** | **Lecturer 1.** “Resident need to read more and they need to improve their English skill.”  **Lecturer 2.** “Some residents they don’t understand clearly the outcome of the course and the amount of lessons and rotation part is too large for them.”  **Lecturer 3.** “Resident need to read more by themself, they need to improve their English skill and learning in scientist such as a reading journal, topic presentation.” | **Resident 1.** “First, we need a standard protocol of surgery in the same rotation place. Second, we need more lecture, a short lecture is fine. Third, a commentator for presentation, we need clear explanation. Not just only student have to read by themselves.”  **Resident 2.** “First, we need more lecture running by teacher, even it short lecture or long lecture. we need more interaction lecture between teacher and resident more than self-study.”  **Resident 3.** “Lao’s textbooks and more guideline on surgical surgery would help us in learning.” |
| **CIPP** | **Content** | **Learning outcome overlapped** | **Lecturer 1.** “No overlapped due to difference technical they use in 4 main hospitals’ Second, we need more update resource of studying.”  **Lecturer 2.** “Not overlapped but depend on each preceptor in each hospital teaching style.”  **Lecturer 3.** “I think the lesson is moving from the basics to the practice and transmits the lesson may be different by each teacher, but it’s not overlapped.” | **Resident 1.** “In my opinion, some is overlapped. We have to study likes all in one class including resident and intern. The lesson should be separate between two group, the resident should be deeper than intern, intern is more basic than us about anatomy. What I mean is we already study that before, but we still study the same thing with intern twice.”  **Resident 2.** “In my opinion, some is overlapped. But I think the basic or theory and practice lesson is totally difference. First, we learn basic part, but when we rotate to hospital, we learn the same thing but deeper in practice.”  **Resident 3.** “I think some of class was overlapped for example teacher leached the same lesson in first year and second year we able to learn that gain in lecture time .” |
|  |  | **Difficulties in maintaining course** | **Lecturer 1.** “Time consuming is difficult because the lecture has work overload in hand.”  **Lecturer 2.** “We maintain by having a monthly meeting one a month and also meeting with 4 main hospital and meeting with resident every 3 months.”  **Lecturer 3.** “We have discussion in a monthly faculty meeting, about teaching and learning , rotation in hospital, discussing about the resident now, how are they working. And also, preceptor meeting and resident meeting every 3 months.” | **Resident 1.** “I heard that they have a monthly meeting between faculty and meeting of the OB-GYN department with the four main hospitals, moreover we have our meeting with coordinator every three months to discuss what difficulty we face on our rotation in each hospital and what we want to learn more about in the course.”  **Resident 2.** “I’m not sure about faculty meeting but we have residents meeting with coordinator every three months to discuss what difficulty we face on our rotation in each hospital, what is our weakness, and we interest to learn more.”  **Resident 3.** “Maybe teacher have their own meeting, as we are resident, we have our meeting with coordinator every three months to discuss about our lesson and rotation.” |
|  | **Input** | **Classroom or meeting room** | **Lecturer 1.** “Not enough anymore, we have to borrow or book a lecture room in four main hospitals.”  **Lecturer 2.** “During covid-19 we used ZOOM, this day if we don’t have available room, we have to borrow or book a lecture room in one of the four main hospitals.”  **Lecturer 3.** “The classroom is not sufficient. We have to borrow a lecture room from one of the four main hospitals or using ZOOM sometimes.” | **Resident 1**. “We have to borrow one of the four main hospitals in advanced or moving to second campus but the distant is quite far and this’s a reason why some residents don’t want to join the lecture.”  **Resident 2.** “No, it’s not enough. If we don’t have available room, the class should be cancelled sometime. We need a fix place for lecture if possible.”  **Resident 3**. “We have difficulty in borrow the classroom, no fix class for each year.” |
|  |  | **Skill lap or practise place** | **Lecturer 1.** “We have one, but equipment is not enough.”  **Lecturer 2.** “We have a skill lap but it’s small and the equipment is not sufficient, resident can’t use, only practice with real patient.”  **Lecturer 3.** “We have a skill lap, but the equipment is not sufficient and the room now under the contraction, resident can’t use, we use real practice with patient instead of this.” | **Resident 1.** “Since COVID19 we never learning practice with mannikin, and the equipment is not sufficient. We only practice with real patient in the hospital.”  **Resident 2.** “No, we don’t have. Since COVID19 we are using ZOOM for learning and only practice in the hospital with real patient. We would like to have practice with mannikin.”  **Resident 3** “We never practice in skill lab, only practice with patient in the hospital.” |
|  |  | **Staff or assistance** | **Lecturer 1.** “The number of staff is enough in quantity but in skill is not that sufficient.”  **Lecturer 2.** “Coordinator is sufficient in the number but not sufficient in quantity, the number of residents is over than the staff.”  **Lecturer 3.** “Even we have a clinical preceptor to help but we need an extra 4-5 staff because we have to expand the residents between 4 main hospitals, so we need more staff to keep an eye on them.” | **Resident 1.** “In my case, , I have one coordinator that we can contact any time, he understands our case and help us everything such as doing a practice and running a lecturer. But some didn’t want to help.”  **Resident 2** “I think not enough, but even the coordinator is not enough in each hospital but some coordinator really doing a good work, we don’t have a difficultly to contact with them.”  **Resident 3.** “I think the staff is enough in each hospital, but we can contact a few of them, some of them didn’t want to contact with us.” |
|  | **Process** | **Collect the opinion of the student** | **Lecturer 1.** “Less of the lecturers collect the student’s opinion.”  **Lecturer 2.** “We don’t have this, but we use the feedback from resident when we have a meeting every three month.”  **Lecturer 3.** “In my case, I did the survey after teaching resident every three months by using a google form, focus on practice more than theory.” | **Resident 1.** “No, we don’t have. But we have that before starting the class, professor will give us a question and teach us, after the class we have to answer him and he will summary lesson again. And we have a feedback practice in each hospital after our rotation using a google form.”  **Resident 2**. “No, we have only feedback practice in each hospital after our rotation by using a google form.”  **Resident 3.** “No, don’t have. We only have some feedback when we conduct a meeting with coordinator.” |
|  | **Product** | **Learning outcome had been achieved** | **Lecturer 1.** “Achieved but not reach 100% because resident knowledge level is not equal due to difference background of living. One more thing, we can see that we succeeded reduce maternal mortality since we started the OB-GYN course in 2003.”  **Lecturer 2.** “80% of learning outcome were reached. We trained residents and doctors who would return to their hometowns but lack of specialists in some provinces, they are unable to perform difficult operation due to lack of staff and equipment.”  **Lecturer 3.** “Because we don’t have any follow-up or evaluation after graduated before. We used to evaluate by going for teaching Basic and Comprehensive Emergency Obstetric Care in the province and see what they can do. We found that most of them can fix the emergency case such as shoulder dystocia ,breech delivery, preterm labor and also reduce the fetal-maternal death.” | **Resident 1.** “This training has been quite beneficial to me. We can continue to explore, allowing us to catch up with real patients, we can make a diagnosis, and find effective papers for reference. I saw our senior who finished the course, some of them became a clinical preceptor, demonstrate that they can use what they've learned to help or teach us.”  **Resident 2.** “This OB-GYN training has been quite beneficial us, we know how to diagnosis, give a treatment and take care of patient. We can use in our real carrier because we practice a lot with real patient.”  **Resident 3.** “Thec ourse really benefit to my needed, I can see and give a treatment and take care of patient in real workplace. We can use in our real carrier because we practice a lot with real patient.” |
